# The reliability of FEbrile Neutropenia after ChEmotherapy (FENCE) scores in predicting granulocyte colony-stimulating factor breakthrough febrile neutropenia among patients with lymphoma undergoing first-cycle chemotherapy: A prospective observational study

**DOI:** 10.3389/fmed.2023.1122282

**Published:** 2023-03-13

**Authors:** Pravinwan Thungthong, Supat Chamnanchanunt, Tawatchai Suwanban, Chajchawan Nakhahes, Kunapa Iam-arunthai, Tananchai Akrawikrai, Udomsak Bunworasate, Ponlapat Rojnuckarin

**Affiliations:** ^1^Division of Hematology, Department of Medicine, Rajavithi Hospital, College of Medicine, Rangsit University, Bangkok, Thailand; ^2^Department of Clinical Tropical Medicine, Faculty of Tropical Medicine, Mahidol University, Bangkok, Thailand; ^3^Division of Hematology, Department of Medicine, Faculty of Medicine, Chulalongkorn University, Bangkok, Thailand; ^4^Research Unit in Translational Hematology, Faculty of Medicine, Chulalongkorn University, Bangkok, Thailand

**Keywords:** lymphoma, FENCE score, cancer, adult, first cycle chemotherapy

## Abstract

**Background:**

A tool for estimating risk of febrile neutropenia (FN) after chemotherapy, namely the FEbrile Neutropenia after ChEmotherapy (FENCE) score, has been developed but has not been widely validated. This study aimed to validate the FENCE score as a tool for predicting granulocyte colony-stimulating factor (G-CSF) breakthrough FN among patients with lymphoma who underwent chemotherapy.

**Methods:**

This was a prospective observational study of treatment-naive adult patients with lymphoma who underwent their first cycle of chemotherapy between 2020 and 2021. The patients were followed up until the next cycle of chemotherapy to identify any infection events.

**Results:**

Among the 135 patients with lymphoma, 62 (50%) were men. In a comparison of the value of each FENCE parameter for predicting G-CSF breakthrough infection, the parameter of advanced-stage disease showed high sensitivity of 92.8%, and receipt of platinum chemotherapy showed high specificity of 95.33%. With a FENCE score of 12 as a cutoff for low risk, analysis across all patients with lymphoma resulted in a high AUROCC of 0.63 (95% CI = 0.5–0.74%; *p* = 0.059), and analysis across only patients with diffuse large B-cell lymphoma (DLBCL) resulted in an AUROCC of 0.65 (95% CI = 0.51–0.79%; *p* = 0.046). With a cutoff point of 12, FENCE score can predict breakthrough infection events at 30.0% (95% CI = 17.8–47.4%).

**Conclusion:**

This study divided patients with lymphoma into risk groups according to FENCE score, showing that this instrument has discriminatory ability in predicting FN events, these being more likely to occur in patients in the intermediate- and high-risk groups. Multicenter studies are needed to validate this clinical risk score.

## Introduction

Infection after chemotherapy commonly occurs in patients with hematologic malignancies who have undergone chemotherapy. This is an emergency condition, causing morbidity and mortality. Infection prevention is the key to improving survival rates among patients with lymphoma. The incidence rate of neutropenic infection after chemotherapy is 10–50% among all patients with malignancies and more than 80% among patients with hematologic malignancies (1, 2). Thus, prophylaxis with granulocyte colony-stimulating factor (G-CSF) is widely used among patients with lymphoma, according to the incidence of patients with lymphoma is 20% with specific risk factors related to physical age, poor performance status, and other comorbidities (3, 4). However, there are cases of G-CSF breakthrough infection among patients with hematologic malignancy undergoing chemotherapy (5–7).

A risk prediction model may help guide hematologist physicians to increase intervention to minimize G-CSG breakthrough infection. Prognostic risk assessment was introduced by the National Comprehensive Cancer Network (NCCN) Clinical Practice Guidelines in Oncology for Hematopoietic Growth Factors, which define intermediate risk as a 20% probability of developing an infection after chemotherapy regimens (4). Primary prophylaxis with G-CSF injection can reduce the risk of infection among patients undergoing chemotherapy regimens who are at intermediate risk, according to NCCN guidance. However, the NCCN guidelines cannot be used to identify individual patients who are at risk. Therefore, the FEbrile Neutropenia after ChEmotherapy (FENCE) score was developed to predict the likelihood of infection on an individual basis (8, 9). Primary prophylaxis with G-CSF alone or in combination with antibiotics for the prevention of chemotherapy-induced infection among patients with lymphoma is a subject of controversy. The first cycle of chemotherapy is important in predicting treatment-related morbidity and mortality. The FENCE score has been validated for the prediction of infection events among patients with malignancy. It has shown moderate discriminatory ability in clinical prediction; the factors included in calculating the score are age, gender, malignancy type, stage, albumin, total bilirubin, estimated glomerular filtration rate, infection events before chemotherapy, and chemotherapy regimen (8). Previous studies have included patients with lymphoma as a small group. This study aimed to validate a model for predicting breakthrough FN in patients receiving G-CSF prophylaxis for their first cycle of chemotherapy based on pre-therapy risk factors in consecutive treatment-naive patients with lymphoma.

## Materials and methods

### Study design and patient enrollment

All patients with lymphoma making initial visits at the Rajavithi Hospital in Bangkok, Thailand, were assessed for inclusion in this prospective observational study of consecutive treatment-naive patients. This study was approved by the Ethical Committee of Rajavithi Hospital (number 083/2021). Patients were enrolled after providing informed consent. The participating patients underwent their first cycle of chemotherapy between 2020 and 2022, with the last follow-up in September 2022. Patients with lymphoma undergoing first-line chemotherapy as appropriate were enrolled in the study. The baseline was defined as the first date of chemotherapy. All patients received G-CSF (either filgrastim or filgrastim biosimilar) as a primary prophylaxis.

An FN event was defined as a culture (regardless of whether any specimen cultures were positive or negative) or death during the development of a neutrophil count <0.5 × 10^9^/L or decline within the next 48 h with a temperature more than 38.3°C. Temperature measurements were routinely performed during hospital visits. Patients' individual FENCE scores were calculated based on the coefficients specified for use in FENCE score calculation (8). Subsequently, each individual's total FENCE score was categorized as low-risk (score < 16), intermediate-risk (score 17–35), high-risk (score 36–52), or very high-risk (score > 53). The FENCE score was calculated from data collected before chemotherapy and follow-up data on the patients until their second cycle of chemotherapy.

### Statistical analysis

The performance of the FENCE score was assessed in two scenarios to test the applicability of our definition of FN: (1) using the definition of FN (i.e., documented fever and neutropenia), and (2) considering only those FN events that met the neutropenia criterion of the definition of FN. A cross-table was constructed for each FENCE parameter indicating its value in predicting FN events. The proportion of all patients with a positive FENCE parameter who experienced an FN event was analyzed to establish measures such as sensitivity, specificity, and negative predictive value for all FENCE parameters; these are reported in the form of percentages with a 95% confidence interval (CI). For likelihood ratios, the log-likelihood method was used to calculate a percentage with 95% CI. A comparison was considered significant if the two-sided *p*-value was <0.05.

FENCE score performance was tested in the validation FN cohort, and the discriminatory ability of the FENCE score model was analyzed in terms of the area under the receiver operating characteristic curve (AUROCC) and in terms of incidence rate ratios with 95% CIs for each parameter of the FENCE score. The ROCC is a plot of sensitivity against 1—specificity for each risk parameter. Statistical analysis was performed using SPSS version 18.0 (Mahidol license) and MedCalc version 2022. A *p*-value of <0.005 was taken to indicate statistical significance.

## Results

During the period of the prospective study, 135 chemotherapy-naive patients with lymphoma underwent their first cycle of chemotherapy. Most patients with lymphoma were men (54.1%). The median age of the patients was 59 years. A total of 102 patients were diagnosed with DLBCL (75.6%) and 67 presented with B symptoms (49.6%). Patients who had high LDH at diagnosis were significantly more likely to experience FN (91.4%) than not (*p* < 0.001). Similarly, patients with FN who had ECOG 2 (22.9%) were significantly more than those without FN who had ECOG 2 (3.0%) after the first cycle of chemotherapy. The median duration of G-CSF was 6 days among patients who developed G-CSF prophylaxis breakthrough FN and 3 days among patients who did not develop FN (Table 1). Among all patients with lymphoma, 28 (20.7%) developed G-CSF prophylaxis breakthrough FN, of whom the majority (53.6%) were at low risk based on their FENCE score. There was a significant difference in the proportion of patients falling into the FN (16.1%) and non-FN groups (83.9%; *p* = 0.043) among patients with a low-risk FENCE score. This study observe a 40.0% incidence rate of FN among patients with lymphoma who were categorized as high risk, and a 29.7% incidence rate among those who were categorized as intermediate risk.

**Table 1:** Characteristics of patients with lymphoma (*n* = 135) according to whether they developed febrile neutropenia (FN).

Characteristic^*^	**Developed FN**		***p*-value**
	**Present**	**Absent**	
Age (years)^**^	56.0 (49.0–72.0)	58.9 (50.3–70.0)	0.941
Male	18 (51.4)	44 (44.0)	0.287
Body weight (kg)^**^	53.6 (47.0–61.2)	58.6 (49.0–67.8)	0.101
Body mass index (kg/m^2^)	20.2 (18.1–25.6)	22.8 (20.4–26.5)	0.71
ECOG			0.001
0	7 (20.0)	36 (36.0)	
1	20 (57.1)	61 (61.0)	
2	8 (22.9)	3 (3.0)	
B symptoms			0.022
Present	23 (65.7)	44 (44.0)	
**Comorbid diseases**			
Present	21 (60.0)	51 (51.0)	0.236
**HBsAg test**			
Positive	1 (2.9)	11 (11.0)	0.13
**Lymphoma type**			
DLBC lymphoma	25 (71.4)	77 (77.0)	0.328
Other lymphoma	10 (28.6)	23 (23.)	
**LDH at diagnosis**			
High	32 (91.4)	61 (61.0)	< 0.001
Bone marrow involvement	7 (20.0)	12 (12.0)	0.185
**Extra-nodal involvement**			
Absent	20 (57.1)	38 (38.0)	0.039
Hemoglobin^**^ (g/dL)	11.5 (9.1–13.1)	11.6 (10.3–13.3)	0.41
White blood cells^**^ (/uL)	8,570 (5,570–10,280)	7,430 (5,640–11,240)	0.568
Platelet count^**^	305.0 (210.0–370.0)	293.0 (227.7–361.7)	0.544
**Chemotherapy regimen**			
CHOP	8 (22.9)	26 (26.0)	0.275
R-CHOP	11 (31.4)	43 (43.0)	
Other	16 (45.7)	31 (31.0)	
Duration of G-CSF (days)	6 (4–6)	3 (2–6)	0.606
**FENCE score**			
Low (score < 16)	15(16.1)	78 (83.9)	0.043
Intermediate (score 17–35)	11 (29.7)	26 (70.3)	
High (score 36–52)	2 (40.0)	3 (60.0)	

* Number (percentage).

** Median; IQR.

The FENCE parameter of disease stage (advanced stage) had a high sensitivity of 92.86% (95% CI = 76.50–99.12%). Receipt of chemotherapy with non-platinum alkylating agents, topoisomerase, antimetabolites, and vinca alkaloids, also showed a sensitivity of 89.29–82.14%. The parameter with the highest specificity was receipt of a chemotherapy regimen with platinums regimen (95.33%; 95% CI = 89.43–98.47%). Albumin count (hypoalbuminemia) had a specificity of 83.18 (95% CI = 74.72–89.71%) (Table 2).

**Table 2:** Accuracy of each FENCE parameter in predicting FN events.

**Parameter**	**Gender**	**Age**	**Cancer type**	**Disease stage**	**Albumin**	**Bilirubin**	**CKD-EPI**	**Platinum**	**Non-platinum alkylating agents**	**Topoisomerase**	**Antimetabolites**	**Vinca alkaloids**
**Negative results**												
n/N	48/62	63/82	24/33	41/43	89/104	19/25	68/87	102/127	1/4	12/16	1/5	5/10
%	77.4	76.8	72.2	95.3	85.6	76.0	78.2	80.3	25.0	75.0	20.0	50.0
**Sensitivity**												
n/N	14/28	9/28	19/28	26/28	13/28	22/28	9/28	3/28	25/28	24/28	24/28	23/28
% (95% CI)	50.0 (30.65–69.3)	32.14 (15.88–52.35)	67.88 (47.65–84.12)	92.86 (76.50–99.12)	46.43 (27.51–66.13)	78.57 (59.05–91.70)	32.14 (15.88–52.35)	10.71 (2.27–28.23)	89.29 (71.77–97.73)	85.71 (67.33–95.97)	85.71 (67.33-95.97)	82.14 (63.11-93.94)
**Specificity**												
n/N	48/107	63/107	24/107	41/107	89/107	19/107	68/107	102/107	1/107	12/107	1/107	5/107
% (95% CI)	44.86 (35.23–54.78)	58.88 (48.95–68.30)	22.43 (14.93–31.51)	38.32 (29.08–48.22)	83.18 (74.72–89.71)	17.76 (11.04–26.33)	63.55 (53.69–72.64)	95.33 (89.43–98.47)	0.93 (0.02–5.10)	11.21 (5.93–18.77)	0.93 (0.02-5.10)	4.67 (1.53-10.57)
**NPV**												
% (95% CI)	77.42 (69.13–84.0)	76.83 (71.07–81.74)	72.73 (58.36–83.45)	95.35 (84.07–98.76)	85.58 (80.62–89.43)	76.0 (58.29–87.77)	78.16 (72.76–82.74)	80.31 (78.09–82.36)	25.0 (3.48–75.51)	75.0 (51.16–89.58)	20.0 (2.83-68.25)	20.74 (14.25-28.56)
**NLR**												
% (95% CI)	1.11 (0.73–1.71)	1.15 (0.85–1.58)	1.43 (0.75–2.73)	0.19 (0.05–0.72)	0.64 (0.45–0.92)	1.21 (0.53–2.73)	1.07 (0.8–1.43)	0.94 (0.82–1.07)	11.46 (1.24–106.05)	1.27 (0.44–3.65)	15.29 (1.78-131.42)	3.82 (1.19-12.28)
**PLR**												
% (95% CI)	0.91 (0.60–1.36)	0.78 (0.44–1.4)	0.87 (0.66–1.15)	1.51 (1.26–1.80)	2.76 (1.55–4.93)	0.96 (0.77–1.18)	0.88 (0.49–1.60)	2.29 (0.58–9.02)	0.90 (0.79–1.03)	0.97 (0.82–18.77)	0.87 (0.74-1.01)	0.86 (0.72-1.03)
**Accuracy**												
% (95% CI)	45.93 (37.32–54.71)	53.33 (44.56–61.96)	31.85 (24.10–40.42)	49.63 (40.92–58.36)	75.56 (67.42–82.54)	30.37 (22.76–38.87)	57.04 (48.24–65.52)	77.78 (69.82–84.48)	19.26 (12.96–26.93)	26.67 (19.43–34.96)	18.52 (12.36-26.11)	20.74 (14.25-28.56)

NPV, negative predictive value; NLR, negative likelihood ratio; PLR, positive likelihood ratio.

**Figure 1:**
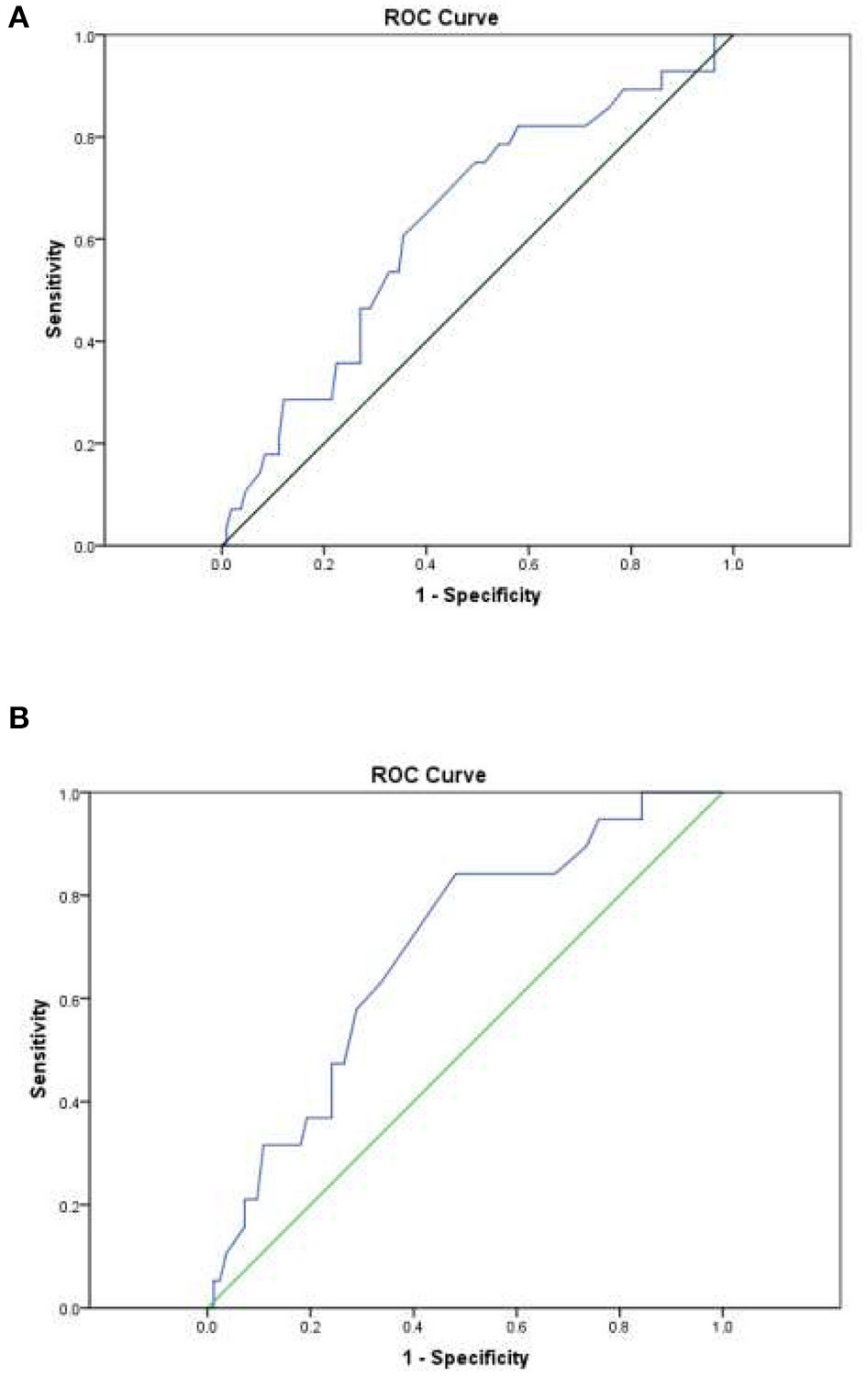
Area under the receiver operating characteristic curve (AUROCC) for individual patient FENCE score as used in prediction of febrile neutropenia. **(A)** Across all lymphoma patients: AUROCC = 0.63 (95% CI = 0.52–0.75; *p* = 0.028). **(B)** Among DLBCL patients only: AUROCC = 0.69 (95% CI = 0.57–0.82; *p* = 0.009).

Total FENCE score was used to classify patients into one of the following FENCE groups: low-risk (total score < 16), intermediate-risk (total score 17–35), or high-risk (total score 36–52). The AUROCC across all patients with lymphoma was 0.63 (95% CI = 0.52–0.75%; *p* = 0.028). The AUROCC for the sub-group of patients with DLBC was 0.69 (95% CI = 0.57–0.82%; *p* = 0.009) (Figure 1). Among all patients with lymphoma, we compared low-risk patients with intermediate- and high-risk patients because of the small number of high-risk patients. In terms of identification of the most suitable cutoff point for FENCE score, use of a cutoff point of 16 for the total FENCE score resulted in a sensitivity of 46.43% (95% CI = 27.51–66.13%) and specificity of 72.90% (95% CI = 63.45–81.04%). Higher sensitivity (64.29%; 95% CI = 44.07–81.36%) was observed in the test over all patients with lymphoma when a cutoff point of 12 was used as the FENCE score designating the threshold for low risk, compared with other potential cutoff points. High specificity (65.42%; 95% CI = 55.61–74.35%) was observed in the test over all patients with lymphoma when a cutoff point of 14 was used as the FENCE designating the threshold for low risk. Focusing on only patients with DLBC, the use of a cutoff point of 16 for the total FENCE score resulted in a sensitivity of 47.37% (95% CI = 24.45–71.14%) and specificity of 75.90% (95% CI = 65.27–84.62%). Among this group of patients, high sensitivity (63.16%; 95% CI = 38.36–83.71%) was observed when a cutoff point of 12 was used, and high specificity (72.29%; 95% CI = 61.38–81.55%) was observed when a cutoff point of 14 was used to designate low risk (Table 3).

**Table 3:** Accuracy of total FENCE score in predicting FN events.

**Parameter**	**All patients with lymphoma (*n = 135)***				**DLBC patients only (** ***n = 102)***			
	**Low-risk cutoff set to 16**	**Low-risk cutoff set to 14**	**Low-risk cutoff set to 12**	**Low-risk cutoff set to 10**	**Low-risk cutoff set to 16**	**Low-risk cutoff set to 14**	**Low-risk cutoff set to 12**	**Low-risk cutoff set to 10**
**Negative results**								
n/N	78/93	65/75	65/75	53/74	63/73	60/69	55/62	37/53
%	83.9	86.7	86.7	71.6	86.3	87.0	88.7	71.2
**Sensitivity**								
n/N	13/28	15/28	18/28	7/28	9/19	10/19	12/19	4/19
% (95% CI)	46.43 (27.51–66.13)	53.57 (33.87–72.49)	64.29 (44.07–81.36)	25.00 (10.69–44.87)	47.37 (24.45–71.14)	52.63 (28.86–75.55)	63.16 (38.36–83.71)	21.05 (6.05–45.57)
**Specificity**								
n/N	78/107	70/107	65/107	53/107	63/83	60/83	55/83	37/83
% (95% CI)	72.90 (63.45–81.04)	65.42 (55.61–74.35)	60.75 (50.84–70.05)	49.53 (39.72–59.37)	75.90 (65.27–84.62)	72.29 (61.38–81.55)	66.27 (55.05–76.28)	44.58 (33.66–55.90)
**NPV**								
% (95% CI)	83.87 (78.33–88.21)	84.34 (77.95–89.13)	86.67 (79.45–91.62)	71.62 (64.45–77.08)	86.30 (80.17–90.75)	86.96 (80.29–91.60)	88.71 (81.05–93.52)	71.15 (63.85–77.50)
**NLR**								
% (95% CI)	0.73 (0.51–1.06)	0.71 (0.47–1.08)	0.59 (0.35–0.99)	1.51 (1.14–2.02)	0.69 (0.45–1.08)	0.66 (0.40–1.07)	0.56 (0.30–1.02)	1.77 (1.27–2.47)
**PLR**								
% (95% CI)	1.71 (1.03–2.84)	1.55 (1.01–2.39)	1.64 (1.14–2.35)	0.50 (0.25–0.97)	1.97 (1.07–3.61)	1.90 (1.10–3.29)	1.87 (1.19–2.96)	0.38 (0.16–0.93)
**Accuracy**								
% (95% CI)	67.41 (58.81–75.22)	62.96 (54.23–71.11)	61.48 (52.72–69.72)	44.44 (35.90–53.24)	70.59 (60.75–79.20)	68.63 (58.69–77.45)	65.69 (55.63–74.81)	40.20 (30.61–50.37)

NPV, negative predictive value; NLR, negative likelihood ratio; PLR, positive likelihood ratio.

**Figure 2:**
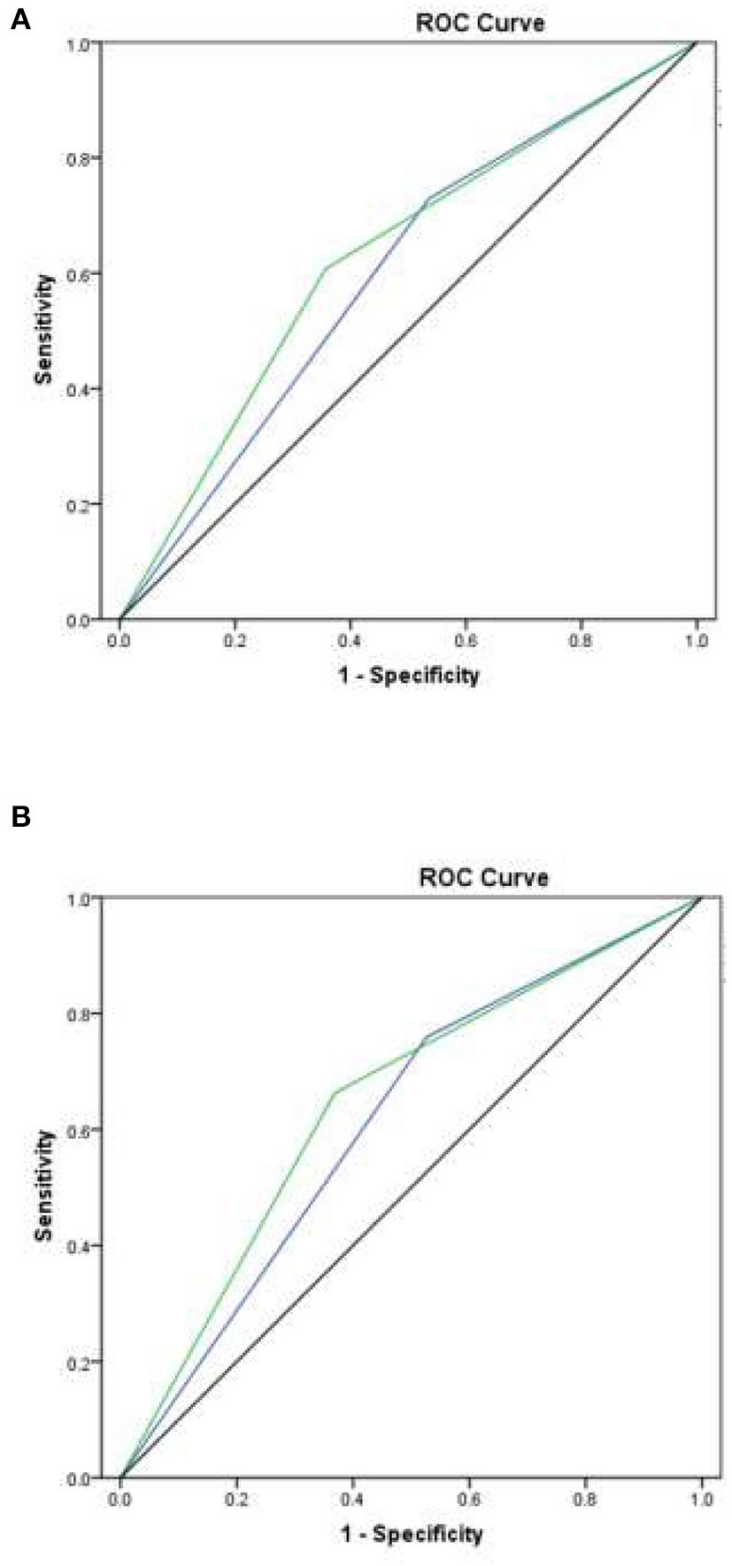
Area under the receiver operating characteristic curve (AUROCC) for individual patient FENCE score as used in prediction of febrile neutropenia, taking different cutoff values. **(A)** Across all lymphoma patients: with a low-risk cutoff of 16 (blue line), AUROCC = 0.59 (95% CI = 0.47–0.72; *p* = 0.062); with a low-risk cutoff of 12 (green line), AUROCC = 0.63 (95% CI = 0.51–0.74; *p* = 0.059). **(B)** Among DLBC lymphoma patients only: with a low-risk cutoff of 16 (blue line), AUROCC = 0.62 (95% CI = 0.47–0.76; *p* = 0.075); with a low-risk cutoff of 12 (green line), AUROCC = 0.65 (95% CI = 0.51–0.79; *p* = 0.046).

**Figure 3:**
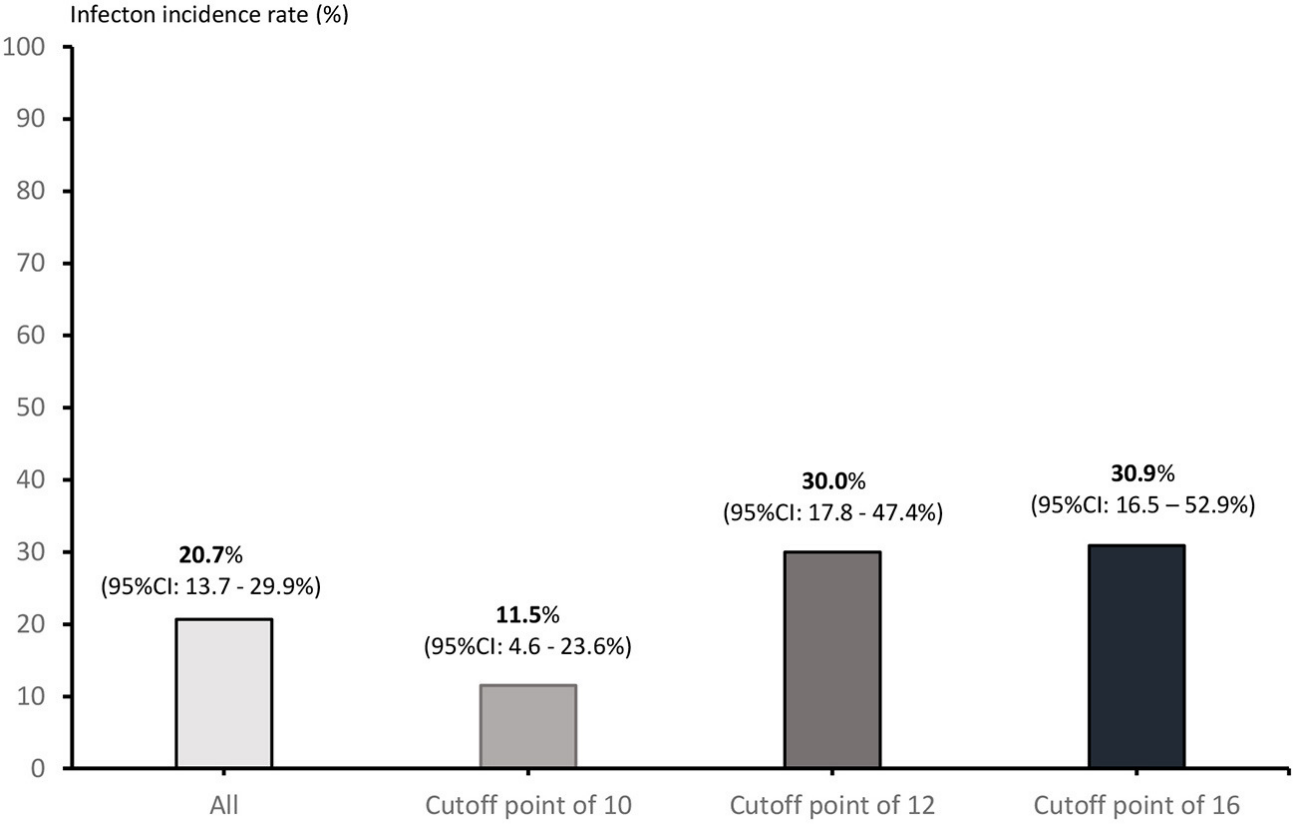
Incidence of G-CSF prophylaxis breakthrough FN events according to each possible cutoff point.

Across all patients with lymphoma, the use of a cutoff point of 16 for low risk resulted in an AUROCC of 0.59 (95% CI = 0.47–0.72%; *p* = 0.062); the use of a cutoff point of 12 for low risk resulted in an AUROCC of 0.63 (95% CI = 0.51–0.74%; *p* = 0.059). Among the sub-group of patients with DLBCL, the use of a cutoff point of 16 for low risk resulted in an AUROCC of 0.62 (95% CI = 0.47–0.76%; *p* = 0.075); the use of a cutoff point of 12 for low risk resulted in an AUROCC of 0.65 (95% CI = 0.51–0.79%; *p* = 0.046) (Figure 2). We reclassified FENCE score at a cutoff of 12, which can predict breakthrough FN events at 30.0% (95% CI = 17.8–47.4%) among patients with lymphoma with G-CSF as primary prophylaxis, 30.9% (95% CI = 16.5–52.9%) at a cutoff point of 16, and 11.5% (95% CI = 4.6–23.6%) at a cutoff point of 10, compare to patients with DLBCL with G-CSF breakthrough infection was 20.7% (95% CI = 13.7–29.9%) (Figure 3).

## Discussion

Our study assessed methods of classifying treatment-naive adult patients with lymphoma into risk groups based on FENCE score according to the risk of G-CSF prophylaxis breakthrough FN in the first cycle of chemotherapy. This score demonstrated a moderate discriminatory ability for the prediction of FN events. Specifically, use of the low-risk FENCE score cutoff as a threshold resulted in good discriminatory ability in predicting FN in this study (8, 9). The incidence of FN among patients with lymphoma undergoing their first cycle of chemotherapy was consistent with other studies (4, 9–13). This study included patients with lymphoma, with a median age of 59 years, who received G-CSG prophylaxis. However, there are a few other types of DLBCL, which potentially limits the reproducibility and generalizability of the findings with respect to G-CSF prophylaxis.

The discriminability of each FENCE parameter suggested the importance of advanced-stage disease and of receipt of an EPOCH regimen (non-platinum alkylating agents, topoisomerase, antimetabolites, or vinca alkaloids); this finding can aid in determining individual parameters predicting the development of FN (4). This may result in discrepancies in use of the overall FENCE score as a risk parameter because patients with advanced-stage disease received high-intensity chemotherapy, which is considered a risk factor for developing FN. The factors with the highest specificity in prediction of FN were hypoalbuminemia and receipt of platinum chemotherapy, which is similar to the standard guidelines (4, 14). Platinum-based chemotherapy is the most common form of chemotherapy (8). Patients with lymphoma should be closely monitored and promptly receive supportive care.

Aagaard et al. developed a risk group classification based on total FENCE score. Their study combined cases of solid tumor and hematologic malignancy (lymphoma) (8). On this basis, our study used a cutoff point of < 16 to identify low-risk patients, which resulted in only 46.43% sensitivity and 72.90% specificity in prediction of FN events. The low-risk score was related to G-CSF prophylaxis breakthrough infection. Patients with lymphoma with a total FENCE score >16 (i.e., those falling into the intermediate- and high-risk groups) might need close monitoring of fever after receiving chemotherapy. The NCCN guidelines for hematopoietic growth factors define an intermediate risk of developing FN or infection after chemotherapy as a probability of infection events of over 20% (4, 15–17). Our findings indicated scores of 12 and 16 as potential cutoff points for the prediction G-CSF prophylaxis breakthrough FN at ~30% probability of infection events. Extended routine use of G-CSF prophylaxis and close monitoring for infection are beneficial for these groups. Our study indicated that 12 would be a more appropriate cutoff point for the low-risk group, as the use of this cutoff resulted in high sensitivity and specificity. Similarly, analysis of the sub-group of patients with DLBCL also demonstrated an optimal cutoff point of 12 for the low-risk group, resulting in high sensitivity and specificity. Across all patients, the AUROCC value among all patients with lymphoma at a cutoff point of 12 than at a cutoff of 16. Notably, only patients with DLBCL also demonstrated higher values at a cutoff point of 12 than at a cutoff point of 16 of the FENCE score. We postulated the cutoff point of 12 of the FENCE score for the low-risk group, which showed differences in the patients' populations such as only patients with lymphoma and chemotherapy regimen (not including taxanes drug). This may influence the discrepancies in the FENCE risk group.

Our cohort study of FENCE risk groups is the first to analyze only patients with lymphoma. The limitations of the FENCE risk group classification in this cohort are as follows. First, the cohort patients with lymphoma included a small group of patients without DLBCL (such as those with T-cell lymphoma), who underwent a different form of chemotherapy. Second, patients underwent chemotherapy regimens composed of different drugs, which the original FENCE score counts as only a single chemotherapy drug for the purpose of calculating the total score. Third, patients received G-CSF prophylaxis at different times relative to the start of their chemotherapy. This might affect the prevention of G-CSF breakthrough infection, and some patients might miss their daily dose of G-CSF (6, 18). Finally, patients with lymphoma who received targeted therapy (such as rituximab) would be considered as having received “other chemotherapy” for the purpose of calculating the FENCE score. Targeted therapy might not increase the risk of FN among patients with lymphoma based on the guidelines (4). To our knowledge, this is the first prospective observational study to validate the FENCE score model in patients with lymphoma. The FENCE score is applicable to patients with lymphoma because it relies on individual risk factors that we routinely record. The discriminatory ability and implementation of the FENCE score in clinical practice may guide hematologist physicians in identifying patients who are most likely to benefit from monitoring for G-CSF prophylaxis breakthrough infection.

## Conclusion

This study showed that patients with lymphoma who underwent chemotherapy with G-CSF prophylaxis developed G-CSF prophylaxis breakthrough infections. Those patients with a FENCE score indicating that they are at risk might benefit from a combination of G-CSF and antibiotic prophylaxis. Further prospective multicenter studies are needed to identify the range of FENCE scores indicating intermediate or higher risk.

## Data availability statement

The datasets presented in this article are not readily available because the data that supported these findings of a study are available on request from the corresponding author. The data are not publicly available due to privacy and ethical restrictions. Requests to access the datasets should be directed to supat.cha@mahidol.ac.th.

## Ethics statement

This study was approved by the Ethical Committee of Rajavithi Hospital (Number 083/2021). The patients/participants provided their written informed consent to participate in this study.

## Author contributions

PT and SC contributed to conception and design of the study, performed the statistical analysis, and wrote the first draft of the manuscript. PT, SC, TS, CN, KI-a, and TA organized the database. PT, SC, UB, and PR wrote and commented sections of the manuscript. All authors contributed to manuscript revision, read, and approved the submitted version.
